# *In-vitro* Callus Induction and Rosmarinic Acid Quantification in Callus Culture of *Satureja khuzistanica* Jamzad (Lamiaceae)

**Published:** 2014

**Authors:** Amir Sahraroo, Mesbah Babalar, Mohammad Hossein Mirjalili, Mohammad Reza Fattahi Moghaddam, Samad Nejad Ebrahimi

**Affiliations:** a *Department of Horticulture, Faculty of Agriculture, University of Tehran, Karaj, Iran.*; b *Department of Agriculture, Medicinal Plants and Drugs Research Institute, Shahid Beheshti University, G. C., Evin, Tehran Iran.*; c *Department of Phytochemistry, Medicinal Plants and Drugs Research Institute, Shahid Beheshti University, G. C., Evin, Tehran, Iran.*

**Keywords:** *Satureja* sp, Lamiaceae, Callus culture, Rosmarinic acid, HPLC UV/MS

## Abstract

In the present study, an efficient protocol has been developed for callus induction and production of RA in callus culture of *Satureja khuzistanica* for the first time. *In-vitro* callus induction was achieved from young shoot tip explants cultured on MS and B5 media supplemented with different concentrations of IBA (0.1, 1.0, 2.0 and 5.0 mgL^-1^) solely or in combination with cytokinins BAP and KIN (1.0, 2.0 and 5.0 mgL^-1^). B5 medium supplemented with 1.0 mgL^-1^ IBA plus 5.0 mgL^-1^ BAP and MS medium fortified with 2.0 mgL^-1^ IBA and 2.0 mgL^-1^ BAP were the most favorable media for callus formation with the highest induction rate (96%). Maximum growth index (2.89 and 2.63) and maximum callus biomass (2.34 and 2.33 g fresh weight) were obtained from the callus cultured on B5 medium supplemented with 1.0 mgL^-1^ IBA plus 5.0 mgL^-1^ BAP and MS medium fortified with 1.0 mgL^-1^ IBA plus 1.0 mgL^-1^ KIN, respectively. Determination and quantification of RA in cultured calli were performed by HPLC UV/MS analysis. Calli induced from the plant and maintained on supplements of IBA and BAP in the absence of light produced RA 7.5% based on dry weight (DW). No differentiation was observed in any callus during the course of this study.

## Introduction

**Figure 1:**
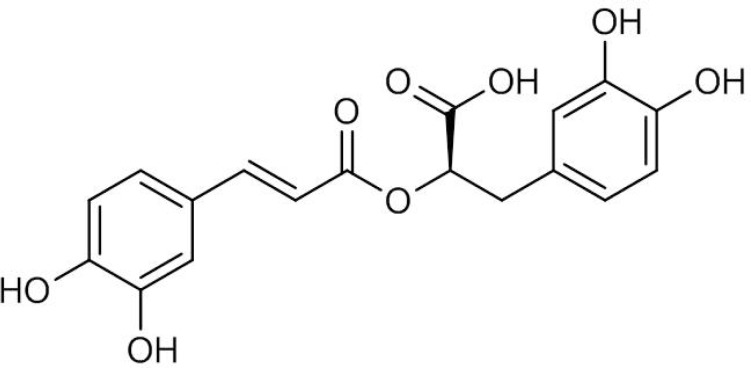
Chemical structure of rosmarinic acid (RA).

Rosmarinic acid (RA) is a water-soluble phenolic acid (Figure 1) which is mainly found in the plant species of Lamiaceae and Boraginaceae (1). RA derived from caffeic acid and (*R*)-(+)-3-(3, 4-dihydroxyphenyl) lactic acid represents one of the most common caffeic esters in plant material which was originally identified in *Rosmarinus officinalis *L. and accumulated constitutively (2). RA possesses various biological activities such as antimicrobial, anti-inflammatory, antimutagenic, improvement of cognitive performance, prevention of the development of Alzheimer’s disease, cardioprotective effects, reduction of the severity of kidney diseases, antioxidant and cancer chemoprevention (3, 4). RA is also used commercially for food preservation. There are many products on the market containing RA, however, only recently the pure compound has been used as a commercial drug. These features outline it as an interesting product for both pharmaceutical and cosmetic industries. 

With an increasing demand for the bioactive products, the plant cell cultures provide an attractive alternative source that could overcome the limitations of extracting useful metabolites from limited natural resources. Plant cell cultures are also widely employed as a model system to investigate the production of specific secondary metabolites such as phenolic acid derivatives because they offer experimental advantages both to basic and applied research and to the development of models with scale-up potential (5-9). Additionally, the commercial cultivation of plant cells represents an alternative source of secondary metabolites, helping to save the genetic diversity of the wild population of medicinal plants (10). Various studies have been previously carried out for the production of RA in callus and cell suspension cultures of some Lamiaceae members such as *Solenostemon blumei* (Benth.) M. Gomez (syn.: *Coleus blumei*) (11), *Salvia officinalis* (12, 13), *Lavandula vera* (14), *Agastache rugosa* (15), and *Ocimum sanctum* (16). Although generation of callus and suspension culture of *Satureja hortensis* has been described (17), there is no information available in literature with respect to other *Satureja* species. During our ongoing efforts to investigate natural sources of phenolic acid derivatives, we found a potent unique plant species, *S. khuzistanica* growing in the Southwest of Iran as endemic species (18). Realizing the importance of the plant and its extracts in clinical medicine (19, 20), we attempted to apply this strategy to establish callus culture of the plant acting as source for the production of RA. The present work investigated the ability of RA production in a callus culture of *S. khuzistanica*. 

## Experimental

*Chemicals and reagents*

Basal media salts, vitamins, sucrose, polyvinylpyrrolidone (PVP), L-glutamine, casein hydrolysate, agar, plant growth regulators (PGRs), HPLC grade methanol and standards of RA were purchased from Merck (Darmstadt, Germany) and Sigma (Sigma-Aldrich Corporation, Spruce Street, St. Louis, MO, USA). HPLC grade water was used throughout the analysis. 

*Plant material*

Stem cuttings of *S. khuzistanica* were collected from wild-growing plant populations in Khuzistan Province (33° 00′ 334″ N, 47° 40′ 999″ E at an altitude of 490 m) in the Southwest of Iran. They were then rooted in the greenhouse for further sampling. Shoot segments were excised from newly produced shoots of rooting plants growing in the greenhouse condition (24 ± 2 ºC/15 ± 2 ºC day/night) and were used as explants. A voucher specimen of *S. khuzistanica* (MPH-1582) has been deposited at the Herbarium of Medicinal Plants and Drugs Research Institute (MPH), Shahid Beheshti University, Tehran, Iran.

*Decontamination of shoot segments*

Shoot segments of the plant were washed thoroughly under running tap water for 30 min, then soaked in 70% ethanol for 30 s, and decontaminated by a 10 min with 1% (v/v) commercial bleach (5% sodium hypochlorite) containing a few drops of Tween-80, followed by rinses in sterile distilled water. 

*In-vitro shoot tip multiplication*

Shoot segments (3 cm) of *S. khuzistanica* were aseptically inoculated on Murashige and Skoog (MS) medium (21) containing 0.2% (w/v) PVP, 3% (w/v) sucrose, and gelled with 0.75 % (w/v) agar supplemented with 2 mgL^-1^ 6-benzylaminopurine (BAP). The pH was adjusted to 6.0 with either 1 N NaOH or HCl prior to addition of agar and autoclaved (Wisd Co. South Korea) for 20 min at 121 °C (1.4 Kg cm^–2^). The cultures were incubated at 25 ± 2 °C under a 16 h photoperiod, with light provided by cool daylight fluorescent lamps (40 µmol^–1^ m^–2 ^s^–1^), and were proliferated by monthly subcultures to fresh medium of the same type. *In-vitro* multiplied shoots were transferred to the hormone free MS medium and then were used for callus induction. 

*In-vitro callus induction and culture conditions*

*In* *-* *vitro* shoot tips (3 nodes) of the plant were vertically cultured on MS and B5 (22) media containing 20 mgL^-1^ L- glutamine, 0.2% (w/v) PVP, 3% (w/v) sucrose supplemented with various concentrations (0, 1, 2, 5 mgL^-1^) of cytokinins BAP or kinetin (KIN) solely or in combination with 0.1, 1, 2 or 5 mgL^-1^ indole-3-butyric acid (IBA). All the cultures were incubated at 25 ± 2 °C under a 16 h photoperiod, with light provided by cool daylight fluorescent lamps (40 µmol^–1^ m^–2 ^s^–1^). After 28 days inoculation, the ability of the explants to develop callus under various conditions was recorded as the callus induction rate (%) which was defined as: 

Callus induction rate (%) = (Total number of explants produced callus/ Total number of explants cultured) × 100%. Fifteen replicates were used per treatment. The Number of days required for callus induction in each treatment was also recorded. 

*Callus maintenance and biomass determination*

The callus was maintained on the B5 medium containing 0.2% (w/v) PVP, 3% (w/v) sucrose, and gelled with 0.75 % (w/v) agar supplemented with 5 mgL^-1 ^BAP, 1 mgL^-1^ IBA, 20 mgL^-1^ L-glutamine, 100 mgL^-1^ casein hydrolysate, and incubated under dark at 24 ± 2 ºC with subcultures at 4 weeks interval in fresh medium. Growth Index (GI) of callus tissue or increasing value of callus fresh weight was calculated as below formula:

GI = (W_1_-W_0_)/W_0_: where W_0_ was the weight of callus tissue before treatment and W_1_ the final weight of callus after culture period. Biomass was also measured by dry weight (DW). Callus tissues were harvested and washed with distilled water three times to remove any residual medium. Then, the cells were lyophilised (Lyophilizer, CHRiST, Germany) at – 40 °C until a constant weight was achieved.

*Statistical analysis*

The experiment was repeated four times and each repeat had four replicates. Data were analyzed in a factorial based on completely randomized design (CRD). Data were analyzed using statistical programs SAS (*Version 9.1.3*). Statistically significant averages were compared using Duncan’s Multiple Range tests. Differences were regarded as significant at *P ≤ 0.05*.

*Extraction and HPLC UV/ELSD, HPLC UV/MS analysis*

 500 mg of lyophilized and powdered samples were extracted by MeOH by aid of ASE (Accelarted Solvent Extraction). Extracts for the initial screening were prepared with an ASE 200 extraction system with solvent module (Dionex) by consecutive treatment with methanol. Extraction pressure was 120 bar and temperature was set at 70 °C. Three extraction cycles of 5 min; preheated time: 1min; flush: 100% of cell volume; purge 80 s with nitrogen in 11 mL steel cartridge. The extracts of three extraction cycles were combined.

*HPLC- PDA analysis*

HPLC separations were carried out on an Waters Alliance series 2690 system equipped with degasser, binary high pressure mixing pump, autosampler, column thermostat and photodiode array (PDA) detector. Data acquisition and processing was performed using Empower2.0 software. (Waters Cooptation, USA). Separation conditions for the MeOH extracts: SunFire C18 column (3.5 μm, 150 × 3.0 mm I.D., Waters, Milford, MA, USA) equipped with a guard column (20.0 × 3.0 mm I.D.); mobile phase A: H_2_O with formic acid 0.1%, mobile phase B: MeCN, flow rate: 0.4 mL/min, column temperature: 25.0 °C, sample injection volume: 20 μL, solvent composition: 25% B isocratic for 5 min, 25–100% B in 10 min), 100% B isocratic for 5 min. 

*LC–MS instrumentation and conditions*

HPLC separations were carried out on an Agilent series 1100 system equipped with degasser, binary high pressure mixing pump, autosampler, column thermostat and photodiode array (PDA) detector (Agilent Technologies; Waldbronn, Germany). The HPLC was coupled to an Esquire 3000 plus ion trap mass spectrometer equipped with an electrospray (ESI) interface (Bruker Daltonics; Bremen, Germany). Data acquisition and processing was performed using HyStar 3.0 software (Bruker Daltonics). E SIMS spectra were recorded in the negative ion mode under ion charge conditions (ICC 20000), at a scan speed of 13000 *m/z/s*, using a gauss filter width of 0.2 m/z. Nitrogen was used as drying gas at a flow rate of 10 L/min and as a nebulizing gas at a pressure of 30 psi. The nebulizer temperature was set at 300 °C. Spectra were recorded in the range of m/z 150 to 1500. Capillary voltage set at −4500 V, endplate offset at 500 V, capillary exit at −128.5 V, skimmer voltage at −40 V, and trap drive at 61.4 V. Data acquisition and processing were performed using Hystar 3.0 software (Bruker Daltonics). A split ratio of 1:1 was used with the ESI interface.

*Preparation of samples for calibration curve*

The mixture of stock standard solution containing RA 1.0 mg/mL was prepared in dimethyl sulfoxide (DMSO). Working standard solution was prepared by serially diluting the stock standard solution with DMSO. Standard solution was diluted to get the final concentrations of the working samples in the range of 0.5 to 200.0 μg/mL in order to establish the calibration curve. All solutions were filtered through 0.45 μm filters and were stored in a refrigerator at 4 °C until further use. Injection volume used for each analysis was 20 μL. The calibration equation was used to calculate the retrieved concentrations, to determine the linearity of the results and to evaluate the statistical parameters of retrieved concentrations.

## Results and Discussion

**Figure 2:**
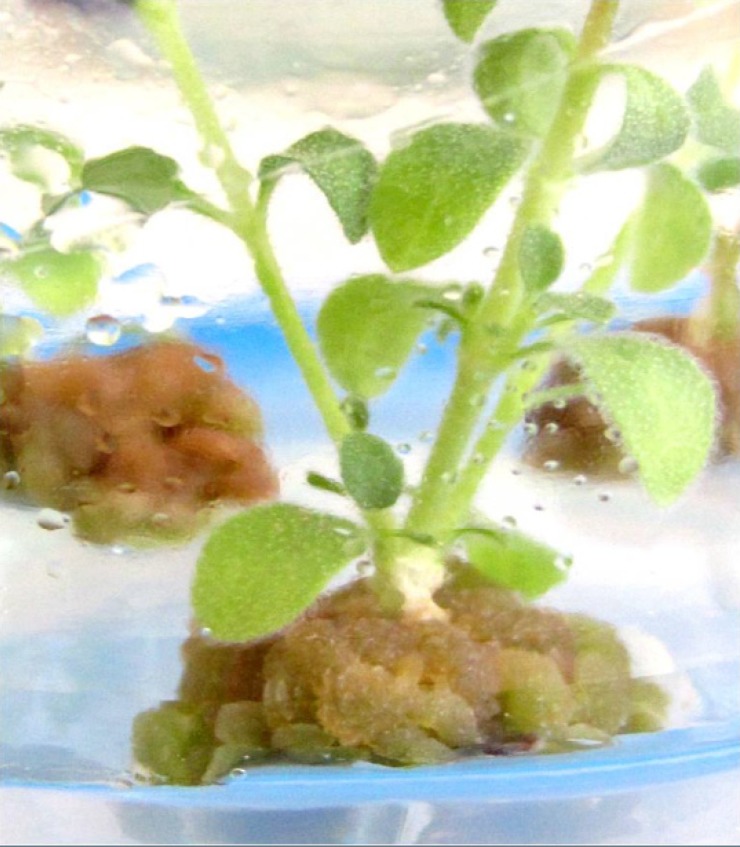
Callus induction at the basal end of *Satureja khuzistanica* shoot tip explants after two weeks of inoculation

A preliminary experiment was carried out using *in-vitro* young leaves, internodes and shoot tip as explants in the callus induction experiment. However, young leaves and internodes cultured with either PGRs or without them began to turn brown after one week of culture. Only shoot tip explants survived and used for further study (data not shown). During the present set of experiment, different PGRs (IBA, BAP and KIN) on MS and B5 media were also tried for callus induction from shoot tip explants. Visible calli were produced at the basal ends of shoot tip explants after two weeks of inoculation (Figure 2). Depending on the PGRs treatments and type of medium culture used, a wide range of variation in frequency of callus formation and nature of callus was observed. No calli were induced in the media without the plant growth regulators (Table 1), indicating that PGRs are required for callus induction. Karam *et al*. (2003) also recorded the same results and emphasized the importance of exogenous PGRs for dividing cells and thus callus formation in *Salvia fruticosa *(23). Our results revealed that the highest percentage of callus induction (96%) was achieved from the B5 medium supplemented by 1 mgL^-1^ IBA + 5 mgL^-1^ BAP as well as MS medium containing 2 mgL^-1^ IBA + 2 mgL^-1^ BAP (Table 1). Similar observations with IBA and BAP, at different concentrations to support callus induction and proliferation were reported earlier in *S. hortensis* (17) and in other Lamiaceae member, *Agastache rugosa *(24). However, optimal concentration of these compounds may depend on many factors, such as a genotype of original plant, explants origin and *etc*. In relation to the callus nature, the calli obtained from B5 medium was friable and white green in color. Friable and light green colored calli were also induced in nearly all media containing 1 mgL^-1^ IBA. In our study, callus biomass progressively increased with an increase in the BAP concentration (Table 2). In this case, the maximum growth index (2.89 and 2.63) and maximum callus biomass (2.34 and 2.33 g fresh weight) were obtained from the callus cultured on B5 medium supplemented with 1.0 mgL^-1^ IBA plus 5.0 mgL^-1^ BAP and MS medium fortified with 1.0 mgL^-1^ IBA plus 1.0 mgL^-1^ KIN, respectively (Table 2). 

**Table 1:** Effect of culture medium and plant growth regulators on callus induction (%) of *Satureja khuzistanica*

**Callus induction (%)**							
**Medium **	**Cytokinin (mgL** ^-1^ **)**		**IBA (mgL** ^-1^ **)**				
			**0**	**0.1**	**1**	**2**	**5**
B5	BAP	1	70^bc^	60^cd^	60^cd^	80^b^	10^h^
		2	80^b^	80^b^	60^cd^	40^ef^	80^b^
		5	10^h^	60^cd^	96^a^	80^b^	80^b^
	KIN	1	20^gh^	80^b^	40^ef^	50^de^	50^de^
		2	70^bc^	30^fg^	50^de^	30^fg^	50^de^
		5	10^h^	80^b^	50^de^	70^bc^	40^ef^
MS	BAP	1	30^fg^	70^bc^	50^de^	70^bc^	50^de^
		2	50^de^	80^b^	50^de^	96^a^	70^bc^
		5	60^cd^	50^de^	80^b^	30^g^	60^cd^
	KIN	1	30^fg^	50^de^	50^de^	50^de^	70^bc^
		2	30^fg^	50^de^	50^de^	60^cd^	50^de^
		5	40^ef^	50^de^	70^bc^	50^de^	60^cd^

Means with different letters are significant according to the Duncan’s multiple range test (P≤0.05).

**Table 2:** Effect of culture medium and plant growth regulators on callus fresh weight of *Satureja khuzistanica*.

**Callus fresh weight (g)**										
				**IBA (mgL** ^-1^ **)**						
				**0**	0.1	1	2		5	
**Medium**		**Cytokinin (mgL** ^-1^ **)**								
B5	BAP		1	1.00^jk^	0.31^stuvwx^	0.23^vwxyz^	1.70^de^			0.67^mno^
			2	0.83^lm^	0.57^nop^	0.83^lm^	0.41^pqrstu^			1.55^ef^
			5	0.03^z^	0.32^rstuvwx^	2.34^a^	2.27^a^			1.32^gh^
	KIN		1	0.04^z^	1.23^hi^	0.45^pqrst^	1.30^gh^			1.42^fg^
			2	0.71^lmn^	0.32^rstuvwx^	0.51^opq^	0.04^z^			0.81^lm^
			5	0.04^z^	1.35^gh^	0.52^opq^	1.25^hi^			0.40^pqrstuvw^
MS	BAP		1	0.28^tuvwxy^	0.54^nopq^	0.40^pqrstuv^	0.85^kl^			0.45^pqrst^
			2	1.84^bcd^	1.872^bc^	0.46^pqrs^	1.72^cd^			1.88^b^
			5	0.22^wxyz^	1.00^jk^	1.99^b^	2.24^a^			0.74^lm^
	KIN		1	0.25^uvwxyz^	0.30^stuvwxy^	2.33^a^	1.55^ef^			1.09^ij^
			2	0.20^wxyz^	0.32^rstuvwx^	0.49^pqr^	0.41^pqrstu^			0.38^qrstuvw^
			5	0.71^lmn^	0.13^yz^	1.11^ij^	0.10^z^			1.95^b^

Means with different letters are significant according to the Duncan’s multiple range test (P≤0.05).

Callus induction and growth of *S. khuzistanica* mostly depended upon the nature of explants as well as the concentration and combination of plant growth regulators, especially cytokinin and auxin. Such types of variation have already been documented for other plant species (25-27). It was observed that the specific ratio of the two hormones significantly increased callus induction and proliferation, which may be due to effects on the endogenous synthesis of the plant growth regulators upon their exogenous application (28). Exogenous application of cytokinin and auxin in a specific ratio may help to maintain the required ratio which favoured callus production. On the other hand, there are several reports concerning the relationship between plant growth regulators and biomass yield of the cultures in the literature. For example, the highest yield in *Salvia officinalis* callus cultures has been observed in the culture medium supported with the equal concentrations of auxin and cytokinin (13). According to a study carried out by Kim *et al.* (2001) on *Agastache rugosa* callus and cell suspension cultures, the highest yield was observed in the culture medium supplemented with 0.5 mgL^-1^ 2,4-dichlorophenoxyacelic acid (2,4-D) and 0.1 mgl^-1^ KIN (15). In another study, no growth was observed in callus, cell suspension and root cultures of *S. fruticosa* in the presence of auxins and cytokinins individually (23).

In the present study, fast growing callus cultures of *S. khuzistanica* were established successfully and maintained for over a period of six months without any change in growth rate. These lyophilized calli were used for extraction and RA quantification. Yamamoto *et al*. (2000) reported that RA was partially decomposed by the lyophilization of cultured *Lithospermum erythrorhizon* cells and was completely decomposed by drying in an oven at 50 °C for 3 days (29). We used a similar method according to Fedoreyev *et al*. (2012) for callus drying (30), considering the technological aspects of using cell biomass containing polyphenols, and analyzed RA by HPLC, according to the procedure described in the Materials and Methods. RA was stable in dry callus biomass (31); therefore, dry cells of *S. khuzistanica* can be used for practical purposes.

**Figure 3:**
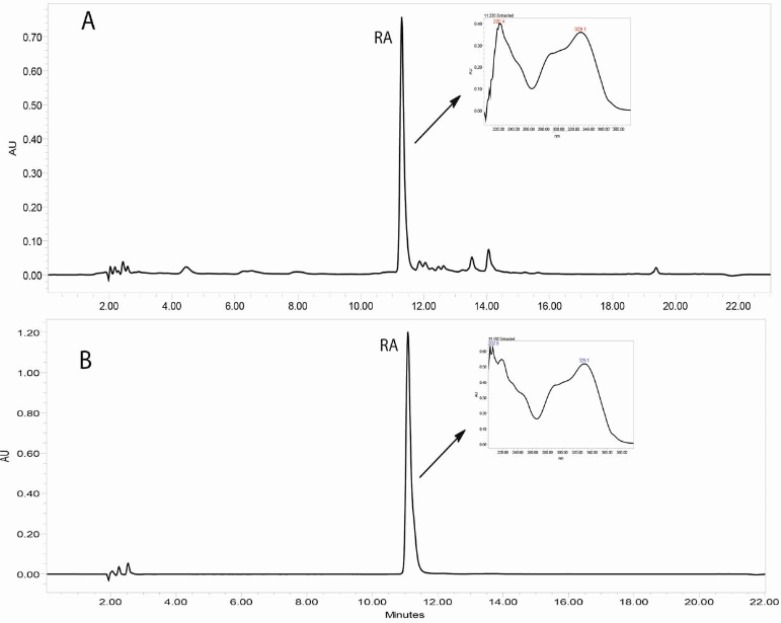
HPLC-UV chromatograms (330 nm) corresponding to the MeOH extract of *in-vitro *callus culture of *Satureja khuzistanica* (A) and the authentic standard of rosmarinic acid (B). Separation conditions involved: Sunfire-RP-C18 column (150 × 3.0 mm, 3.5 μm); mobile phase H_2_O: MeCN, with 0.1% formic acid, flow rate: 0.4 mL/min with an injection volume of 20 μL.

**Figure 4:**
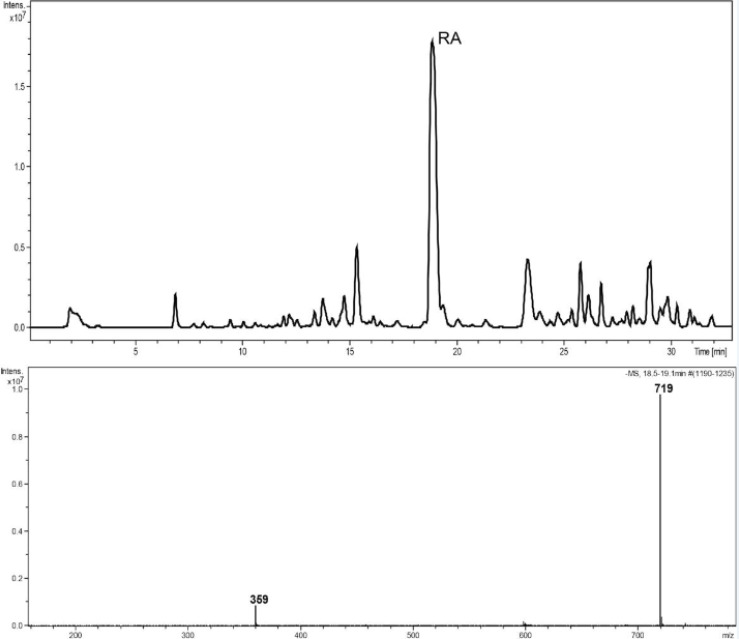
HPLC–ESIMS trace, base peak chromatogram, negative ion mode, m/z 150–1500, MeOH extract of callus culture of *S. khuzistanica*. The molecular weight of 359 is assigned to (M-H)^-^ and 719 to (2M-H)^-^ of rosmarinic acid.

Reversed-phase HPLC-PDA and ESI-MS analysis was performed for the identification of RA in the methanolic extracts of dried callus of *S. khuzistanica* using the authentic compound (Figure 3 and Figure 4) as reference. The content of RA in MeOH extract of callus culture was quantified by HPLC-PDA analysis. Calibration curve for standard of RA showed good linearity at tested concentrations (2 to 200 μg/mL), with correlation coefficients (*r*) of 0.9996. The equation generated from the curve by the external standard method (Table 3) was used to calculate the amount of RA present in crude samples. The amount of RA in dried callus of *S. khuzistanica* was 7.5% DW calculated from the standard equation (Table 3). Our results revealed that the *in-vitro* *S. khuzistanica* callus culture was as effective in producing RA as their mother plant which has been previously reported (18). Based on the results, RA can accumulate in callus cultures to amounts (2.7-fold) much higher than those in intact *S. khuzistanica* plants (2.7% DW).

**Table 3:** Specificity, linearity, sensitivity and content of rosmarinic acid in the callus culture of *Satureja khuzistanica*

Compound	LOD (μg/mL)	LOQ (μg/mL)	Calibration graph	*r*	LDR (μg/mL)	Content (% DW)
Rosmarinic acid	0.02	0.2	y = 133963x + 65868	0.9996	0.2-200	7.5

RA content in some intact Lamiaceae plants has been previously reported by Zgorka and Glowniak (2001) (32). Among the species presented in this report (*Salvia officinalis*, *Melissa officinalis*, *Mentha piperita*, *Thymus vulgaris*, *Lavandula officinalis*, *Rosmarinus officinalis*, *Origanum majorana*, *Hyssopus officinalis*, *Ocimum bacilicum*, and *Satureja hortensis*), maximum RA was determined in *Satureja *species (1.2% DW). RA biosynthesis under *in-vitro* conditions was also confirmed for several species and diverse *in-vitro* culture systems like: callus cultures, suspension cultures, shoot cultures and hairy roots cultures (33, 34). Biosynthesis of RA by *Solenostemon blumei* (syn.: *Coleus blumei*) plant cell culture (35) was the first such process to be announced. Later, it was established that it is also synthesised by other cell cultures such as *Anchusa officinalis* (36), *Lithospermum erythrorhizon* (37), *Orthosiphon aristatus* (38), *Salvia officinalis *(12), *Lavandula vera* (39) and *Satureja hortensis* (17). Kintzios *et al.* (1999) reported that RA content in calli derived from *Salvia fruticosa* and *Salvia officinalis* were 80 and 70 µmol/g fresh weight, respectively (13). Furthermore, Karam *et al*. (2003) demonstrated that the yield of RA was 2.1% DW from callus culture of *Salvia fruticosa *(23). The callus culture of *Ocimum basilicum* accumulated approximately 1 mg RA per gram dry weight and it was slightly more than the concentration in intact plants (40). As far as our literature survey could ascertain, *in-vitro* RA production in *S. khuzistanica* callus cultures have not previously been reported and the present study provides an alternative reproducible protocol for the production of RA as a an important medicinally compound. This protocol can also be contributed to the conservation of this endangered and valuable medicinal species in the wild (41). Its application will facilitate research into the enhanced production of RA and the other phenolic compounds through different biotechnological strategies, like plant cell suspension cultures, and large-scale cultivation in bioreactors.
